# Facile synthesis of hollow Cu_2_O octahedral and spherical nanocrystals and their morphology-dependent photocatalytic properties

**DOI:** 10.1186/1556-276X-7-276

**Published:** 2012-05-30

**Authors:** Lili Feng, Chunlei Zhang, Guo Gao, Daxiang Cui

**Affiliations:** 1 Department of Bio-Nano-Science and Engineering, Research Institute of Micro/Nano Science and Technology, Shanghai Jiao Tong University, Shanghai, 200240, People’s Republic of China

**Keywords:** Cuprous oxide, Crystal growth, Hollow structure, Octahedral nanocrystals, Photocatalysis

## Abstract

Herein, we report that octahedral and spherical Cu_2_O samples with hollow structures are synthesized in high yield by reducing Cu(EDA)_2_^2+^ complex with hydrazine. A series of experiments are carried out to investigate the factors which impact on the morphology of the Cu_2_O samples. It is observed that ethylenediamine (EDA) serves as a molecular template in the formation of hollow structure. Octahedral Cu_2_O with solid structure is prepared without EDA. When EDA is added, Cu_2_O sample with hollow structure is formed. Different morphologies of Cu_2_O such as spherical and octahedral could be obtained by adjusting the concentration of EDA and NaOH. The temporal crystal growth mechanism is proposed. Furthermore, the photocatalytic activities of the as-prepared Cu_2_O nanoparticles are evaluated by monitoring two dyes (methyl orange and congo red) using UV-visible spectrophotometer. Results show that the order of photocatalytic activity of Cu_2_O with different morphologies is as follows: hollow octahedral morphology > hollow sphere morphology > solid octahedral morphology. The hollow octahedral Cu_2_O nanoparticles would be a promising material on applications for photocatalytic degradation of organic pollutants.

## Background

Hollow nanostructures have attracted considerable attention because of their unique physical and chemical properties that allow them to be widely used in catalysts, confined-space chemical reactors [1,2], lithium-ion battery materials [3], controlled gene delivery [4,5], and biomedical diagnosis and therapy [6,7]. Up to date, the hollow structure of nanoparticles is highly desired and typically prepared by sacrificing templates, such as polystyrene, silica, or other inorganic crystals. For example, Yin et al. demonstrated the preparation of hollow CoO nanoparticles (NPs) by the oxidation of Co NPs [8]. Since then, hollow nanocrystals of cobalt oxide and chalcogenides, MnO_2_, metal phosphide, etc. have been prepared through the Kirkendall effect using spherical Co, MnCO_3_, and metal particles as sacrificial templates, respectively [9-11]. Nevertheless, the conventional template approach has intrinsic disadvantages. For example, it is difficult to achieve high yield because of the complicated process. The shell structure may be destroyed in the template removal process if the structure is weak. The conventional template approach is time-consuming, expensive, and complicated in stringent control over a set of experimental variables. Thus, it remains a great challenge to develop feasible methods to prepare hollow nanocrystals with well-defined morphology.

Cuprous oxide (Cu_2_O) is a well-known p-type semiconductor and has a direct small bandgap of 2.2 eV, which endows it promising applications in solar energy conversion [12], as an electrode for lithium-ion batteries [13], gas sensors [14], and photocatalytic degradation of organic pollutants and decomposition of water into O_2_ and H_2_ under visible light [15-17]. So far, great efforts have been devoted to the synthesis of cuprous oxide with different shapes and sizes. Different morphologies such as nanocube, octahedral micro/nanocrystal, and hexapod-shaped microcrystal have been prepared [18-20]. Recently, some methods have been reported on the preparation of cuprous oxide with hollow structure [21-23]. However, these methods mainly focus on spherical morphology formation. Zeng and co-workers [21,22] had prepared hollow Cu_2_O nanospheres and nanocubes through the Ostwald ripening effect using hydrothermal method. However, the preparation of hollow octahedral Cu_2_O nanocrystals usually requires hard templates. Limited reports are closely associated with template-free synthesis of hollow octahedral Cu_2_O nanocrystals. Wang and co-workers [17] and other groups [24] have found that the photocatalytic activity of the [111] surface is much higher than other surfaces, due to higher adsorption capacity on the [111] surface than others (e.g., [100] surface). Cu_2_O with hollow structure and more [111] surface is extensively needed in photocatalysis. Thus, developing effective and facile methods for the synthesis of hollow Cu_2_O crystals especially with hollow octahedral morphology has become a key focus.

In this study, we mainly focused on a facile synthesis of hollow octahedral and spherical Cu_2_O nanocrystals by chemical reduction in which copper salt, sodium hydroxide, ethylenediamine (EDA), and the reducing agent hydrazine hydrate were involved. In this reaction, EDA served as a molecular template in the formation of hollow structure. The morphology of Cu_2_O nanoparticles can be easily tuned from spherical to octahedral with hollow structure by adjusting the concentration of EDA and NaOH. Moreover, the photocatalytic activities of the prepared Cu_2_O nanocrystals were investigated by methyl orange and congo red photodegradation.

## Methods

### Synthesis and characterization of Cu_2_O nanocrystals

All reagents purchased from the Shanghai Chemical Company (Shanghai, China) were of analytical grade and used without further purification. In each synthesis, 20 mL of NaOH (0.1 to 15 mol L^−1^) and varying amounts of EDA (0 to 500 μL; 99 wt.%) were added to a glass reactor (capacity 50 mL). Afterwards, 3 to 10 mL of Cu(NO_3_)_2_ (0.10 mol L^−1^) aqueous solution was added. Followed by a thorough mixing of all reagents, the reactor was then placed in a water bath with temperature controlled over 25°C to 100°C. Finally, hydrazine (50 μL; 35 wt.%) was added to reduce the Cu^2+^ ion to Cu_2_O. After 15 to 60 min, the cuprous oxide products with orange-red color were obtained. The resultant products were washed and harvested with centrifugation-redispersion cycles and dried at 60°C for 4 h in a vacuum oven. Further details on the synthesis can be found in supporting information 1 in Additional file 1.

The crystallographic structure of the products were determined with X-Ray diffraction (XRD) (recorded on a Rigaku D/max-2200/PC (Rigaku Corporation, Tokyo, Japan); test conditions: Cu target at a scanning rate of 7°/min with 2*θ* ranging from 20° to 80°). The morphological investigations of scanning electron microscopy (SEM) images were taken on a field emission scanning electron microscope (FESEM, Zeiss Ultra; Carl Zeiss AG, Oberkochen, Germany). The transmission electron microscopy (TEM) images and electron diffraction patterns of the samples were captured on a JEOL/2100 F transmission electron microscope (JEOL Ltd., Akishima, Tokyo, Japan) at an accelerating voltage of 200 kV.

### Photocatalytic properties of the prepared Cu_2_O nanocrystals

The evaluation of the morphology-related photocatalytic properties of these Cu_2_O samples was performed by constantly monitoring the photocatalytic decolorization of two dyes (methyl orange and congo red) in aqueous solution under visible light irradiation (ordinary household table lamp) by the changes in UV-visible (vis) absorption spectra. The typical procedure was as follows: 0.01 g of the prepared sample was dispersed into 15 mL of the corresponding dye aqueous solution (100 mg L^−1^ for methyl orange; 400 mg L^−1^ for congo red). Before illumination, the suspension was magnetically stirred in the dark for over 2 h to ensure adsorption equilibrium of the corresponding dye on the surface of the Cu_2_O crystal. Then, 300 μL of hydrogen peroxide was added to the solution, and the photocatalytic reaction was carried out with a 40-W daylight lamp (15 cm above the sample) used as a light source (ordinary household table lamp). The corresponding dye aqueous solution was then taken out in 20-, 40-, and 60-min intervals and centrifugated to exclude Cu_2_O. Finally, the corresponding dye aqueous solution was diluted fivefold and measured by a UV–vis spectrophotometer (Varian Cary 50, Varian Inc., Palo Alto, CA, USA).

## Results and discussion

### The morphology of the hollow spherical and octahedral Cu_2_O crystals

**Figure 1:**
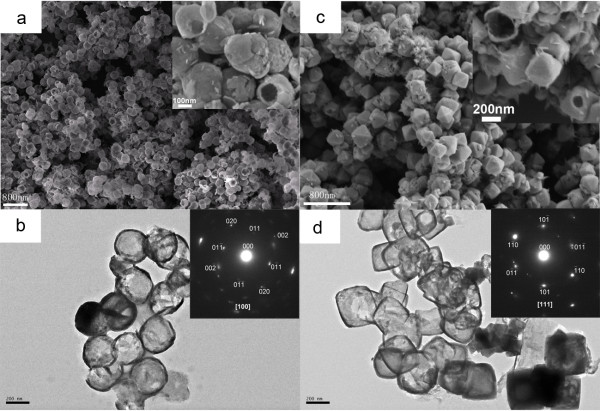
**SEM and TEM images of hollow Cu**_**2**_**O samples.** (**a**, **b**) SEM and TEM images of spherical Cu_2_O (SI1-A1 in Additional file 1), (**c**, **d**) SEM and TEM images of octahedral Cu_2_O (SI1-A2 in Additional file 1). The inset of (a) and (c) are the corresponding enlarged views of hollow Cu_2_O samples. The inset of (b) and (d) are the corresponding SAED patterns of the Cu_2_O samples.

**Figure 2:**
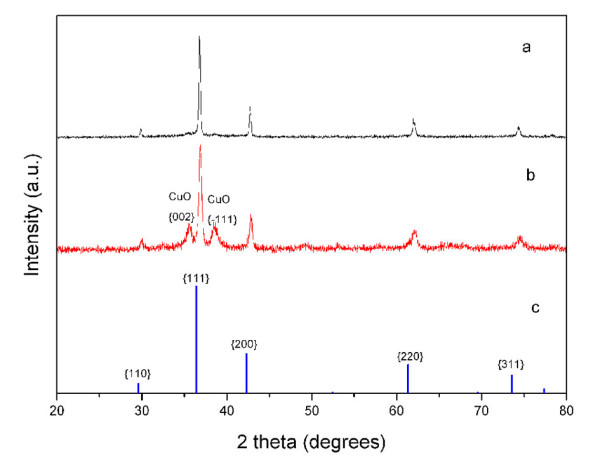
**XRD patterns.** XRD pattern of the (**a**) hollow spherical Cu_2_O (SI1-A1 in Additional file 1), (**b**) octahedral Cu_2_O (SI1-A2 in Additional file 1), and (**c**) JCPDS cards of Cu_2_O (PDF file No.05-0667).

Formation of Cu_2_O hollow crystals is based on the reduction of the Cu(EDA)_2_^2+^ complex by hydrazine. The detailed experimental conditions on the synthesis of hollow spherical and octahedral Cu_2_O crystals were listed in supporting information 1 (A1-A2) in Additional file 1. FESEM and TEM images of Cu_2_O with hollow spherical and octahedral structures were displayed in Figure 1. When the concentration of NaOH was 15 mol L^−1^ and the concentration of EDA was 150 μL/30 mL (the EDA volume was 150 μL; the total volume of this reaction solution was 30 mL; we use 150 μL/30 mL to represent the concentration of EDA), hollow spherical Cu_2_O particles with narrow size distribution were formed. Figure 1a,b showed a panoramic view of the spherical Cu_2_O crystals, which suggested that the product exhibits a uniform, regular shape with an average diameter of about 300 to 350 nm. The crystal orientation and crystallinity of Cu_2_O hollow spheres were further studied with selected area electron diffraction (SAED) methods. The inset of Figure 1b shows the electron diffraction pattern of a Cu_2_O hollow sphere. As indicated by the clear diffraction spots, all of the hollow spheres were nearly single crystalline, although there are a little of intercrystallite mismatches (e.g., splitting of spots, inset of Figure 1b). As for the facets that are exposed, it is speculated that the crystal orientations are [100]. When the concentration of NaOH lowered to 0.1 mol L^−1^ and the concentration of EDA reduced to 40 μL/24 mL, octahedral Cu_2_O crystals with hollows were formed. Figure 1c,d showed the typical morphology of the hollow octahedral Cu_2_O with about 400 nm in size. TEM image showed a hollow structure in the Cu_2_O octahedron. The electron diffraction pattern of a Cu_2_O hollow octahedron was shown in Figure 1d (the inset). The selected area electron diffraction pattern confirms that the particles are single crystals and that the crystal orientation is [111]. XRD analysis was carried out to examine crystallographic structures of the two products (Figure 2). Interplanar distances calculated for [110], [111], [200], [220], and [311] from XRD patterns matched well with Cu_2_O standard data (JCPDS card PDF file no. 05–0667). In the XRD pattern of hollow octahedral samples, very weak diffraction peaks of [002] and [−111] of CuO appeared. As the amount of the reducing agent is many times more than the stoichiometric ratio, CuO should not be formed in the synthesis procedure. CuO might be formed by oxidation of oxygen in water or air in the centrifugation-redispersion procedure. In any case, it proved Cu_2_O crystals were prepared.

### Tuning the morphology of the Cu_2_O crystals by varying the concentration of EDA

**Figure 3:**
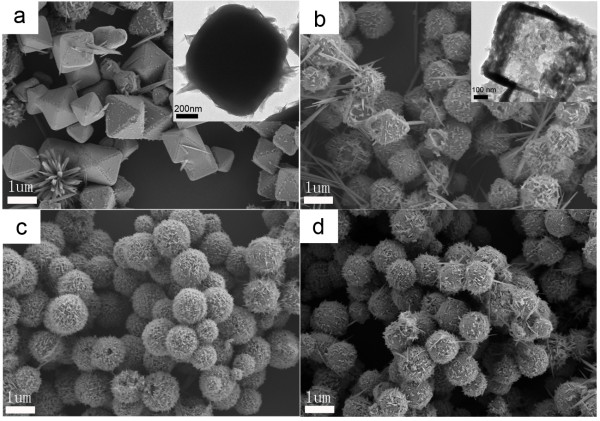
**SEM images of Cu**_**2**_**O samples obtained with different concentrations of EDA.** The concentrations of NaOH solution is 15 mol L^−1^ and the concentrations of EDA were as follows: (**a**) 0 μL (EDA)/28 mL (total solution) (SI1-B1 in Additional file 1), (**b**) 70 μL/28 mL (SI1-B2 in Additional file 1), (**c**) 150 μL/28 mL (SI1-B3 in Additional file 1), and (**d**) 300 μL/28 mL (SI1-B4 in Additional file 1). The inset is the corresponding TEM morphology of the Cu_2_O crystal.

To make out the role played by EDA and other reactants in the procedure and the growth mechanism of hollow Cu_2_O, a series of reactions with different concentrations of EDA in 15 mol L^−1^ and 0.1 mol L^−1^NaOH solutions were investigated. Figure 3 shows the representative SEM images of Cu_2_O prepared with different concentrations of EDA in 15 mol L^−1^NaOH solutions. The detailed experimental conditions were listed in supporting information 1 (B1-B4) in Additional file 1. As shown in Figure 3a, octahedral Cu_2_O crystals about 1,000 nm in size were prepared when EDA was unused. The inset TEM morphology proved the as-prepared Cu_2_O crystals to be a solid structure. The structure of Cu_2_O crystals turned to hollow when the concentration of EDA was 70 μL/28 mL (shown in Figure 3b). When the concentration of EDA continues to increase, the products showed a spherical morphology. From the above results, it is concluded that EDA played a key role on the formation of hollow structure. When EDA is unused, the solid structure was formed. Hollow structure could be formed when EDA was used. XRD results of the four samples were shown in supporting information 2 in Additional file 1. Although the main five diffraction peaks were indexed to Cu_2_O (JCPDS: 05–0667), weak diffraction peaks of Cu for [111] and [211] crystal surfaces (JCPDS: 04–0836) appeared from the solid octahedral sample (Figure 3a) and hollow octahedral sample (Figure 3b). The reason was that hydrazine used as reducing agent is twice the amount of the stoichiometric ratio to reduce Cu^2+^ to Cu_2_O, and some Cu^2+^to Cu.

**Figure 4:**
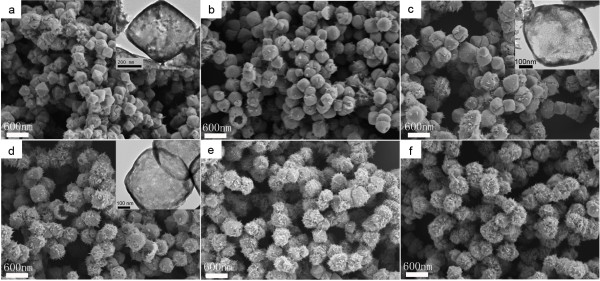
**SEM images of Cu**_**2**_**O samples obtained with different concentrations of EDA.** The concentrations of NaOH solution is 0.1 mol L^−1^ and the concentrations of EDA were as follows: (**a**) 40 μL (EDA)/24 mL (total solution) (SI1-C1 in Additional file 1), (**b**) 70 μL/24 mL (SI1-C2 in Additional file 1), (**c**) 100 μL/24 mL (SI1-C3 in Additional file 1), (**d**) 150 μL/24 mL (SI1-C4 in Additional file 1), (**e**) 200 μL/24 mL (SI1-C5 in Additional file 1), (**f**) 300 μL/24 mL (SI1-C6 in Additional file 1). The inset in SEM is the corresponding TEM morphology of the Cu_2_O crystal.

The morphological evolution of Cu_2_O samples prepared with different concentrations of EDA in 0.1 mol L^−1^NaOH solutions was shown in Figure 4. The detailed experimental conditions were listed in supporting information 1 (C1-C6) in Additional file 1. Under this NaOH concentration, when EDA was unused, precipitation immediately formed. Therefore, EDA is a very important reactant to prevent the formation of Cu(OH)_2_ precipitate, as Cu^2+^ could chelate with EDA. In case NaOH concentration is 15 mol L^−1^, Cu(OH)_4_^2−^ complex could be formed; thus, no precipitation formed when EDA was unused. As shown in Figure 4, hollow octahedral Cu_2_O crystals were prepared when the concentration of EDA was 40 μL/24 mL (shown in Figure 4a). The morphology maintained to be octahedral when the concentration of EDA was from 40 μL/24 mL to 100 μL/24 mL (shown in Figure 4a,b,c). With the concentration of EDA increasing continuously, the morphology turned to spherical (shown in Figure 4d,e,f). Similar to the results of the above experiment (different concentrations of EDA in 15 mol L^−1^NaOH solutions), the concentration of EDA played an important role on the morphological evolution of Cu_2_O. In conclusion, octahedral morphology benefited from the small concentration of EDA, and spherical morphology benefited from the relatively large concentration of EDA. In addition, NaOH concentration is another important factor. When the concentration of NaOH solution was 0.1 mol L^−1^, all the as-prepared products were of uniform hollow structure and about 400 nm in size.

### Growth mechanism

The exact growth mechanism of the hollow Cu_2_O nanocrystals is not very clear. We believe that EDA plays an important role as soft template for hollow structure formation. A classic rolling mechanism inspired by the natural phenomena of a piece of foliage or a piece of wet paper curling into scrolls during its drying process might be applicable for hollow Cu_2_O nanocrystal growth, which was used to explain the formation processes of metal chalcogenide nanotubes and nanorods [25,26]. In that process, lamellar structured intermediates formed firstly. Then, in the reducing course, the lamellar intermediates subsequently rolled up from the edges because sufficient energy was provided to overcome the strain energy barrier. In this stage, nanotubes were usually formed. If the heating time was prolonged, the tubes broke down to afford rod bundles.

**Figure 5:**
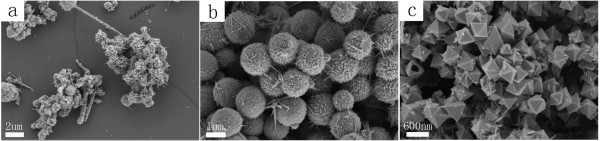
**The formation procedure of hollow cuprous oxide in the presence of EDA.** (**A**) The complex of copper ion, EDA, and hydroxyl. (**B**) The detailed formation procedure of different hollow structure.

In our case, EDA served as bidentate ligand to prevent the formation of Cu(OH)_2_ precipitation and as a molecular template for layered structure formation. The whole formation procedure could be divided into two main stages. In the initial stage, EDA acted as a bidentate ligand, and Cu(EDA)_2_^2+^ complex was formed. Then, the complexes assembled to a layered structure by electrostatic interaction of Cu(EDA)_2_^2+^ and OH^−^ (shown in Figure 5A). In the second stage, the layer-structured Cu(EDA)_2_^2+^ complexes were reduced to Cu_2_O nanolayers after hydrazine was added. Then, the nanolayers rolled up to a hollow structure by the driving force to minimize the surface energy (as shown in Figure 5B). The morphology was related to the detailed experiments and conditions. If the Cu_2_O nanolayers rolled up immediately after hydrazine was added, hollow sphere morphology usually formed. This procedure could be confirmed by some incomplete hollow sphere in Figure 1a. If some Cu_2_O nanolayers firstly self-assembled before being rolled up, a hollow octahedral structure usually formed. The detailed favorable condition for the formation of hollow octahedral structure will be summarized in the following part by more experiments.

### Other factors influenced the morphology of Cu_2_O crystals

**Figure 6:**
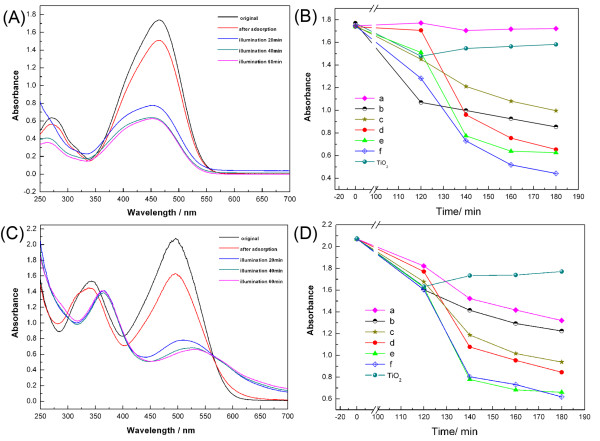
**SEM images of Cu**_**2**_**O samples obtained at different temperatures.** (**a**) 30°C (SI1-D1 in Additional file 1), (**b**) 60°C (SI1-D2 in Additional file 1), and (**c**) 90°C (SI1-D3 in Additional file 1).

In addition to the impact of EDA, other factors also have significant impacts on the morphology. For example, reaction temperature could influence the formation of spherical and octahedral morphologies. Figure 6 is the image of Cu_2_O crystals synthesized at 30°C, 60°C, and 90°C (The detailed experimental conditions were listed in supporting information 1 (D1-D3) in Additional file 1). Cu_2_O with irregular morphology was formed at 30°C. With temperature growth, spherical morphology was formed at 60°C. When reaction temperature reached to 90°C, octahedral Cu_2_O crystals with hollows were formed. Under this synthesis condition (NaOH concentration 15 mol L^−1^), the viscosity of the solution is high; it is difficult to form layer-shaped intermediates at low temperature; thus, irregular morphology was formed at 30°C. When the temperature grew up to 60°C, there was more energy to form Cu_2_O nanolayers and to roll up to hollow sphere. When the reaction temperature reached to 90°C, more energy for the self-assembly of Cu_2_O nanolayers and roll up to octahedral structure was available.

The influence of NaOH concentration on morphological evolution was shown in supporting information 4 in Additional file 1. When NaOH concentration was 0.1 mol L^−1^, the morphology was hollow octahedral. Accompanying with the increase of NaOH concentration, the morphology gradually converted to sphere. When NaOH concentration reached to 5 mol L^−1^, the Cu_2_O crystals were of sphere morphology. Therefore, the results showed that low NaOH concentration was favorable to the formation of hollow octahedral morphology and that high NaOH concentration was favorable to the formation of hollow sphere morphology. Thus, high temperature, low NaOH concentration, and low EDA usage are favorable to the formation of octahedral morphology.

### Photocatalytic activity study

The photocatalytic activities of the Cu_2_O crystals with different morphologies and an TiO_2_ reagent (analytical grade, 100 nm, anatase phase) were evaluated by monitoring the decomposition of two dyes in aqueous solution (methyl orange and congo red) under visible light irradiation. Six samples with different morphologies were studied (shown in supporting information 5 in Additional file 1). Our control experiment showed if only H_2_O_2_ was added in the system, no Cu_2_O, the decomposition of organic pollutants cannot be detected under visible light irradiation (supporting information 6 in Additional file 1). To minimize the influence of adsorption, the photocatalytic experiment was carried out after stirring in the dark for 2 h.

**Figure 7:**
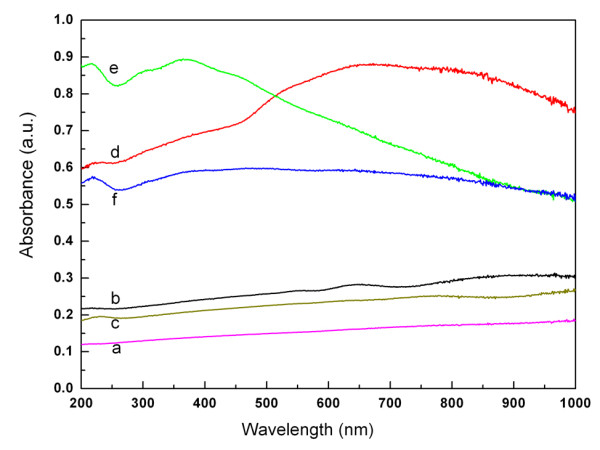
**UV–vis absorption spectra and plots of absorbance versus absorption and irridiation time.** (**A**,**C**) Absorption and photodegradation spectrum of a solution of methyl orange (A) and congo red (C) in the presence of the hollow octahedral Cu_2_O sample shown in Figure1c. (**B**,**D**) Plots of absorbance versus absorption time (0 to 120 min) and irradiation time (20 min, 40 min, 60 min) for methyl orange (B) and congo red (D) in the presence of different Cu_2_O samples: (a), solid octahedral Cu_2_O shown in Figure 3a; (b), hollow sphere Cu_2_O shown in Figure 1a; (c), hollow octahedral Cu_2_O (SI1-D3 in Additional file 1); (d), hollow octahedral Cu_2_O shown in Figure 3b; (e), hollow octahedral Cu_2_O shown in Figure. 1b; (f), hollow sphere Cu_2_O shown in Figure 4d. Analytical grade TiO_2_ reagent (100 nm, anatase phase).

Figure 7A showed the UV–vis absorption spectra of methyl orange (diluted fivefold) photodegraded by hollow octahedral Cu_2_O (SI1-D3 in Additional file 1) at different stages. Figure 7B shows the comparison of photocatalytic activity of different Cu_2_O crystals. Different adsorption abilities of the as-prepared samples were shown at 120-min intervals. Sample *a* (solid octahedral morphology) has the smallest adsorption activity among all the samples. The adsorption activity of sample *d* was only a little higher than sample *a*, and the adsorption activities of samples *a* and *d* were both lower than TiO_2_. Samples *c* and *e* (both hollow octahedral morphology) have the similar adsorption activity to TiO_2_. Samples *f* and *b* (both hollow sphere morphology) have much higher adsorption activity than TiO_2_. The order of adsorption activity of Cu_2_O with different morphologies was as follows: hollow sphere morphology > hollow octahedral morphology > solid octahedral morphology.

After illumination (at 140 min, 160 min, 180 min), TiO_2_ did not show good catalytic activity under visible light irradiation (ordinary household table lamp). Samples *d*, *e*, and *f* with hollow morphology have the highest photocatalytic performance. The decomposition activity of sample *a* (solid octahedral) was the lowest. The order of photocatalytic activity of Cu_2_O with different morphologies was as follows: hollow octahedral morphology with rough surface (samples *d*, *e*, and *f*) > hollow octahedral morphology with smooth surface (sample *c*) > hollow sphere morphology (sample *b*) > solid octahedral morphology (sample *a*).

The photodecomposition results of congo red were shown in Figure 7C and 7D. The adsorption ability of the six samples was similar. The photocatalytic performance of the six samples in the experiment of decomposing congo red followed the similar trends in the experiment of decomposing methyl orange. TiO_2_ did not show good catalytic activity for decomposing congo red under visible light irradiation. Samples *d*, *e*, and *f* have the highest photocatalytic performance. The decomposition activity of samples *a* and *b* was the lowest. However, the photocatalytic performance of sample *a* in the experiment of decomposing congo red is better than that in the experiment of decomposing methyl orange. Morphology-related photocatalytic activities of Cu_2_O followed the similar trends in the experiment of decomposing methyl orange. The order is as follows: hollow octahedral morphology with rough surface > hollow octahedral morphology with smooth surface > hollow sphere morphology > solid octahedral morphology.

In summary, hollow octahedral Cu_2_O had the highest photocatalytic activity to degrade organic pollutants.

### The mechanism of photocatalytic reaction

Cu_2_O has a direct small bandgap of 2.2 eV. The electron can inject from valence band to conduction band when visible light (*λ* > 400 nm) irradiates to the surface of Cu_2_O as shown in Equation 1. The photogenerated electron and holes can rouse a series of reactions as follows [24]: *Reaction (1)* Photogenerated electrons and holes could compound directly in the interior of the Cu_2_O nanomaterial, or the charges can react with Cu_2_O directly; electrons can reduce Cu_2_O to Cu, and holes can oxidize Cu_2_O to CuO. *Reaction (2)* Electrons can reduce H_2_O to H_2_ (Equation 2), whereas holes can’t oxidize H_2_O to O_2_ (Equation 3 because Cu_2_O valence band edge level which is estimated to be +0.6 V is lower than the oxidation potential of water, which is *ca.* +0.82 V at pH 7. *Reaction (3)* The electrons can be scavenged by molecular oxygen O_2_ to yield ·O_2_^−^ (Equation 4), H_2_O_2_ (Equation 5), and OH^−^ (Equation 6). **·**O_2_^−^ and H_2_O_2_ can further inter-react to produce **·**OH (Equation 7). It is well known that the **·**OH is a powerful oxidizing agent with a redox potential of +1.9 V, which can degrade most pollutants (for example, MO can be degraded by **·**OH as shown in Equation 8). *Reaction (4)* If there is some organic compound such as methanol in the solution whose redox potential is more negative than that of CuO/Cu_2_O, the photogenerated holes can oxidize the organics directly.

(1) $${\text{Cu}}_{2}\text{O}+hv\to {\text{e}}^{-}({\text{CBCu}}_{2}\text{O})+h^{+}({\text{VBCu}}_{2}\text{O})$$

(2) $${2\text{H}}_{2}\text{O}+{2\text{e}}^{-}\to {\text{H}}_{2}+{2\text{OH}}^{-}\;\text{E}=-0.41\;\text{V}$$

(3) $${2\text{H}}_{2}\text{O}\to {\text{O}}_{2}+{4\text{H}}^{+}+{4\text{e}}^{-}\;\text{E}=0.82\text{V}$$

(4) $${\text{O}}_{2}+{\text{e}}^{-}\to {\text{O}}_{2}{}^{-}\;\text{E}=-0.28\;$$

(5) $${\text{O}}_{2}+{2\text{H}}_{2}\text{O}+{2\text{e}}^{-}\to {\text{H}}_{2}{\text{O}}_{2}+{2\text{OH}}^{-}\;\text{E}=0.28\text{V}$$

(6) $${\text{O}}_{2}+{2\text{H}}_{2}\text{O}+{4\text{e}}^{-}\to {4\text{OH}}^{-}\;\text{E}=0.40\text{V}$$

(7) $${\text{H}}_{2}{\text{O}}_{2}+{\text{O}}_{2}{}^{-}\to \text{OH}+{\text{OH}}^{-}+{\text{O}}_{2}$$

(8) OH+MO→degradation products

The first reaction usually caused photocorrosion of Cu_2_O, resulting in the loss of photocatalytic activity (reaction 1). In order to prevent the photocorrosion, hole consumption agents such as methanol were usually added to the reaction solution (reaction 4). In our experiment, the reactions were as follows: Firstly, the photogenerated electron and holes were formed by visible light excitation (Equation 1); Secondly, the electrons were scavenged by molecular oxygen O_2_ to yield **·**O_2_^−^ (Equation 4). The **·**O_2_^−^ reacted with H_2_O_2_ to produce **·**OH (Equation 7). Thirdly, the **·**OH was the key oxidizing agent to degrade most pollutants, so the degradation reaction occurred. The addition of H_2_O_2_ in our experiment was very important. It could accelerate the generation of ·OH. In our control experiments, if only H_2_O_2_ was added in the system, no Cu_2_O, the organic pollutants could not be degraded. If only Cu_2_O was added in the system, no H_2_O_2_, the organic pollutants could be degraded with a very slow speed. So, the addition of H_2_O_2_ was essential. TiO_2_ has a wide bandgap of 3.2 eV, so only the shorter wavelength solar energy can be utilized (*λ* < 387 nm). Although TiO_2_ had good performance in photocatalytic reaction under ultraviolet light irradiation [27-29], the photocatalytic property is weak under visible light irradiation. So, the TiO_2_ sample did not have photocatalytic activity in our experiment.

To investigate the reason why hollow octahedral Cu_2_O had the highest photocatalytic activity to degrade organic pollutants, the total surface area test (BET) and the UV–vis absorption spectra of the Cu_2_O samples were done (see Table 1). The total surface area was in the following order: sample *f* > *e* > *d* > *c* > *b* > *a*. It is very similar to the photocatalytic order of Cu_2_O samples (*e* ≈ *f* ≈ *d* > *c* > *b* > *a*). The surface area was the main factor contributing to the photocatalytic activities. Samples *e* (12.0085 m^2^ g^−1^) and *f* (14.0681 m^2^ g^−1^) had a relatively large surface area and were much higher than the literature values 0.1819 m^2^ g^−1^ for the hollow Cu_2_O sample [30]. The hollow Cu_2_O samples made by our method were superior to the literature one. In the BET result, although the total surface area of sample *e* was lower than sample *f*, the photocatalytic activity of sample *e* was higher. This may be ascribed to more [111] surface on sample *e*. In addition, sample *d* has similar photocatalytic activity to samples *e* and *f***,** but they had significant differences in the total surface areas. Here, the UV–vis absorption spectra result will give explanation.

**Table 1:** Total surface area (BET) result

**Sample**	**a**	**b**	**c**	**d**	**e**	**f**
BET(m^2^ g^−1^)	0.0784	1.2535	1.4557	3.8125	12.0085	14.0681

**Figure 8:**
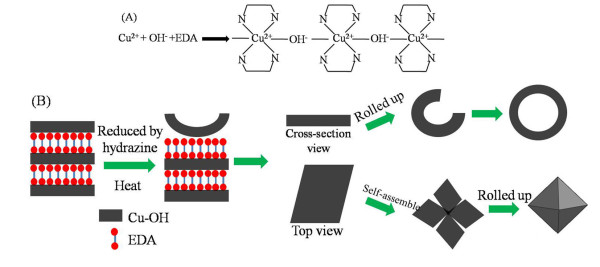
**UV–vis absorption spectra of the Cu**_**2**_**O samples.**

The sample concentration for UV–vis absorption spectra test was 2 × 10^−4^ g mL^−1^ for each. The UV–vis absorption spectra (Figure 8) suggested that the intensities of samples *d*, *e*, and *f* were much stronger. The UV–vis absorption intensities of samples *b* and *c* were lower than samples *d*, *e*, and *f*. Sample *a* had the lowest UV–vis absorption intensities. The order of UV–vis absorption intensities was as follows: samples *d* > *e* > *f* > *c* > *b* > *a*. The photocatalytic activities of Cu_2_O samples obeyed the same order. It is suggested that the photocatalytic activities of the Cu_2_O samples had positive correlation with the UV–vis absorption intensity. Among the six samples, sample *d* had the highest UV–vis absorption intensity, so the utilization efficiency of visible light of sample *d* was higher than sample *f*. It could explain the high photocatalytic activity of sample *d*. In conclusion, apart from the total surface area (the main factor), UV–vis absorption intensity and utilization efficiency of visible light also contributed to the photocatalytic activities.

Finally, different crystal surfaces have significant impact on the catalytic activity. It is reported that the photocatalytic activity of the [111] surface is much higher than other surfaces. Thus, octahedral nanoparticles (mainly coverd by [111] surfaces) have a higher photocatalytic activity than cubic-shaped nanoparticles and spherical nanoparticles (mainly coverd by [100] or [110] surfaces).

In summary, these results demonstrated that the photocatalytic activities of the microcrystals were related to their morphology. The highest photocatalytic activity of hollow octahedral Cu_2_O samples could ascribe to the larger surface area, high utilization efficiency of visible light, and many [111] surfaces.

## Conclusion

In summary, octahedral and spherical Cu_2_O samples with hollow structures had been synthesized by a simple chemical reduction method. In this reaction, EDA played a key role in the formation of hollow structures. Different morphologies of Cu_2_O could be obtained by adjusting the concentration of EDA and NaOH. The photocatalytic activities of these as-prepared Cu_2_O microcrystals with different morphologies, such as solid octahedral, hollow sphere, and hollow octahedral, were investigated by photodegradation of two dyes (methyl orange and congo red). The results demonstrated that hollow octahedral Cu_2_O possessed the highest photocatalytic activity, which could ascribe to the larger surface area, high utilization efficiency of visible light, and many [111] surfaces. The hollow octahedral Cu_2_O would be a promising material on applications for photocatalytic degradation of organic pollutants.

## Competing interests

The authors declare that they have no competing interests.

## Authors’ contributions

LLF designed and carried out the whole study. CLZ and GG participated in the discussion of this research. DXC gave the instruction of the study. All authors read and approved the final manuscript.

## Supplementary Material

Additional file 1 **Supporting information.** A document showing supporting information 1 to 6 for facile synthesis of hollow Cu_2_O octahedral and spherical nanocrystals and their morphology-dependent photocatalytic properties. Click here for file
