# Supplementary material for: Facile synthesis of hollow Cu2O octahedral and spherical nanocrystals and their morphology-dependent photocatalytic properties
Source: Nanoscale Res Lett. 2012 May 30;7(1):276. doi: 10.1186/1556-276X-7-276 (PMC3508613; doi:10.1186/1556-276X-7-276)

**Supporting Information for**

**Facile synthesis of hollow Cu_2_O octahedral and spherical nanocrystals and their morphology-dependent photocatalytic properties**

Lili Feng, Chunlei Zhang, Guo Gao, Daxiang Cui*

Department of Bio-Nano-Science and Engineering, Research Institute of Micro/Nano Science and Technology, Shanghai Jiao Tong University, Shanghai 200240, P. R. China

* Correspondence should be addressed to: [dxcui@sjtu.edu.cn](mailto:dxcui@sjtu.edu.cn)

**Supporting Information 1**

Experimental condition for selected samples in this work:

| **Expt.** | **NaOH aqueous solution** | | **Cu(NO_3_)_2_ aqueous solution** | | **Volume of EDA**  (µL) | **Volume of N_2_H_4_** (35 wt%) (µL) | **Temperature**  ℃ |
| --- | --- | --- | --- | --- | --- | --- | --- |
|  | Volume  (mL) | concentration  mol L^-1^ | Volume  (mL) | concentration  mol L^-1^ |  |  |  |
| **A1** | **20** | **15** | **10** | **0.1** | **150** | **50** | **60** |
| **A2** | **20** | **0.1** | **4** | **0.1** | **40** | **50** | **60** |
|  |  |  |  |  |  |  |  |
| B1 | 20 | 15 | 8 | 0.1 | **0** | 50 | 60 |
| B2 | 20 | 15 | 8 | 0.1 | **70** | 50 | 60 |
| B3 | 20 | 15 | 8 | 0.1 | **150** | 50 | 60 |
| B4 | 20 | 15 | 8 | 0.1 | **300** | 50 | 60 |
|  |  |  |  |  |  |  |  |
| C1 | 20 | 0.1 | 4 | 0.1 | **40** | 50 | 60 |
| C2 | 20 | 0.1 | 4 | 0.1 | **70** | 50 | 60 |
| C3 | 20 | 0.1 | 4 | 0.1 | **100** | 50 | 60 |
| C4 | 20 | 0.1 | 4 | 0.1 | **150** | 50 | 60 |
| C5 | 20 | 0.1 | 4 | 0.1 | **200** | 50 | 60 |
| C6 | 20 | 0.1 | 4 | 0.1 | **300** | 50 | 60 |
|  |  |  |  |  |  |  |  |
| D1 | 20 | 15 | 4 | 0.1 | 150 | 50 | **30** |
| D2 | 20 | 15 | 4 | 0.1 | 150 | 50 | **60** |
| D3 | 20 | 15 | 4 | 0.1 | 150 | 50 | **90** |
|  |  |  |  |  |  |  |  |
| E1 | 20 | **0.1** | 4 | 0.1 | 150 | 50 | 60 |
| E2 | 20 | **1** | 4 | 0.1 | 150 | 50 | 60 |
| E3 | 20 | **5** | 4 | 0.1 | 150 | 50 | 60 |
| E4 | 20 | **10** | 4 | 0.1 | 150 | 50 | 60 |
| E5 | 20 | **15** | 4 | 0.1 | 150 | 50 | 60 |
| E6 | 20 | **1** | 8 | 0.1 | 150 | 50 | 60 |

Note:

Experimental condition of Figure 1(main text): A1-A2.

Experimental condition of Figure 3(main text): B1-B4.

Experimental condition of Figure 4(main text): C1-C6.

Experimental condition of Figure 5(main text): D1-D3.

**Supporting Information 2**

XRD pattern of the Cu_2_O (SI1:B1-B4) samples (The corresponding SEM morphology of the Cu_2_O crystal in Fig. 3 (main text) ).


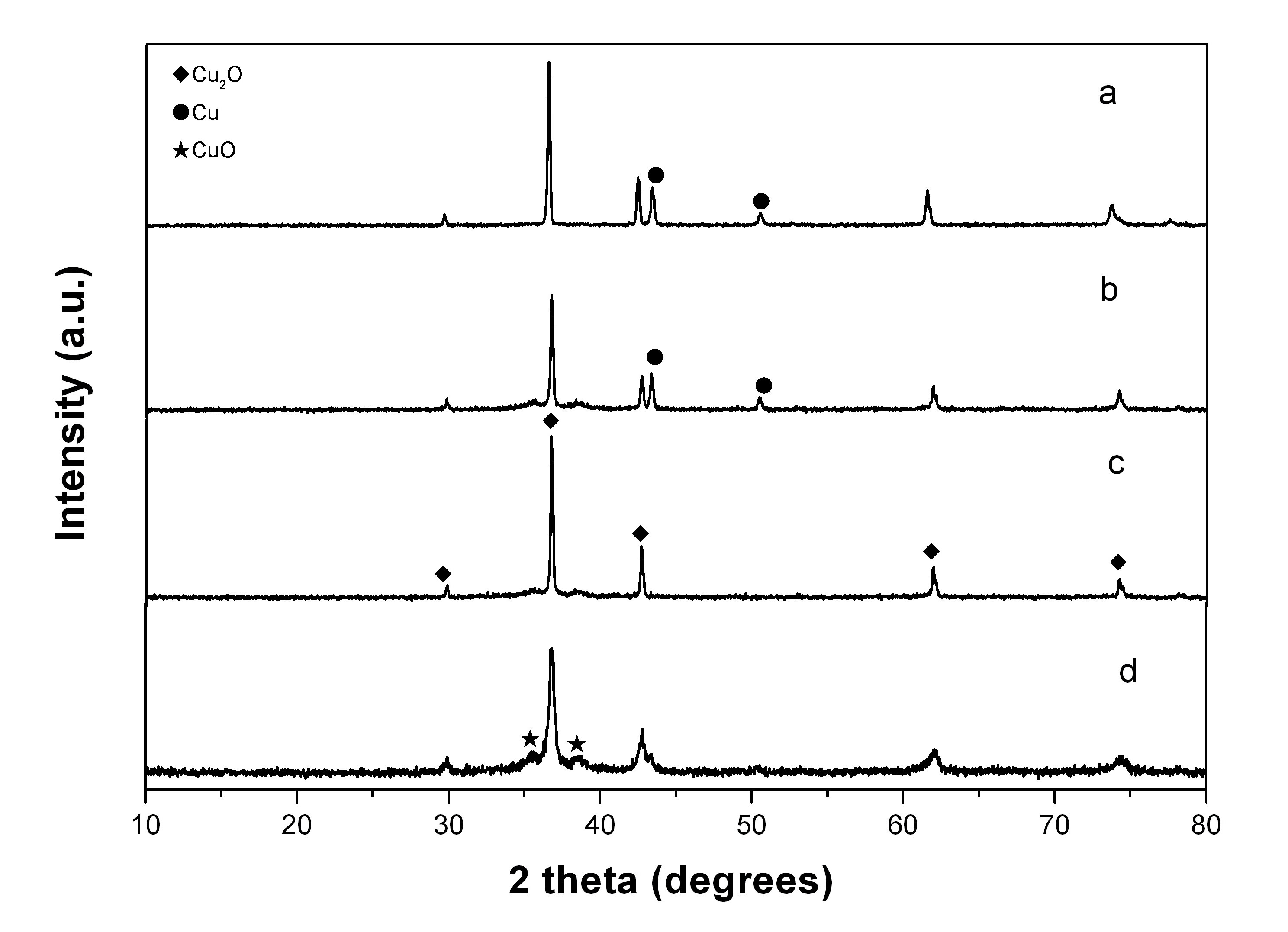


**Supporting Information 3**

XRD pattern of the Cu_2_O (SI1:C1-C6) samples (The corresponding SEM morphology of the Cu_2_O crystal in Fig. 4 (main text) ).


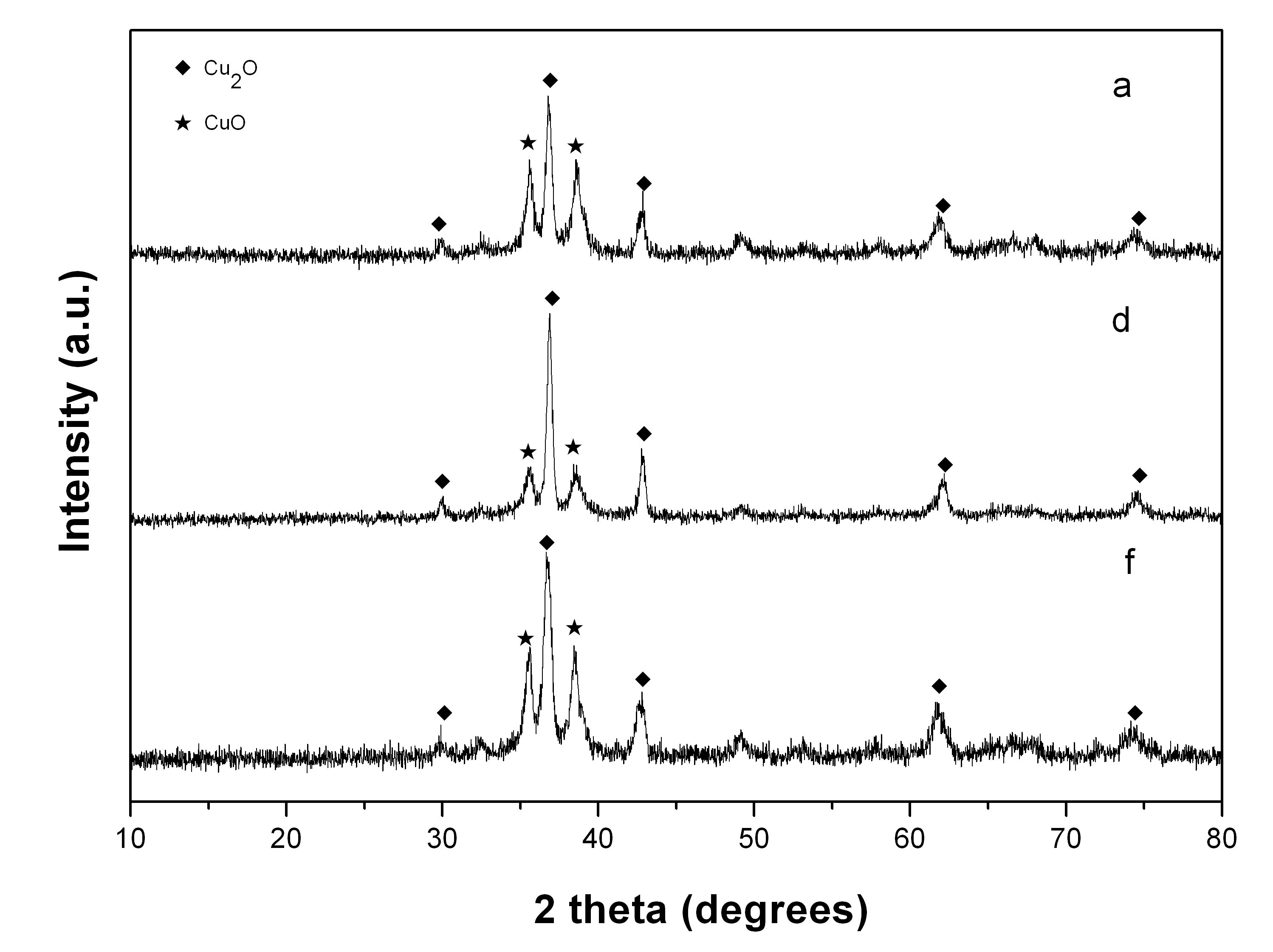


**Supporting Information 4**

SEM images of Cu_2_O samples obtained at different NaOH concentration: (a) 0.1 mol L^-1^ (SI1-E1), (b) 1 mol L^-1^ (SI1-E2), (c) 5 mol L^-1^ (SI1-E3), (d) 10 mol L^-1^ (SI1-E4), (e) 15 mol L^-1^ (SI1-E5)


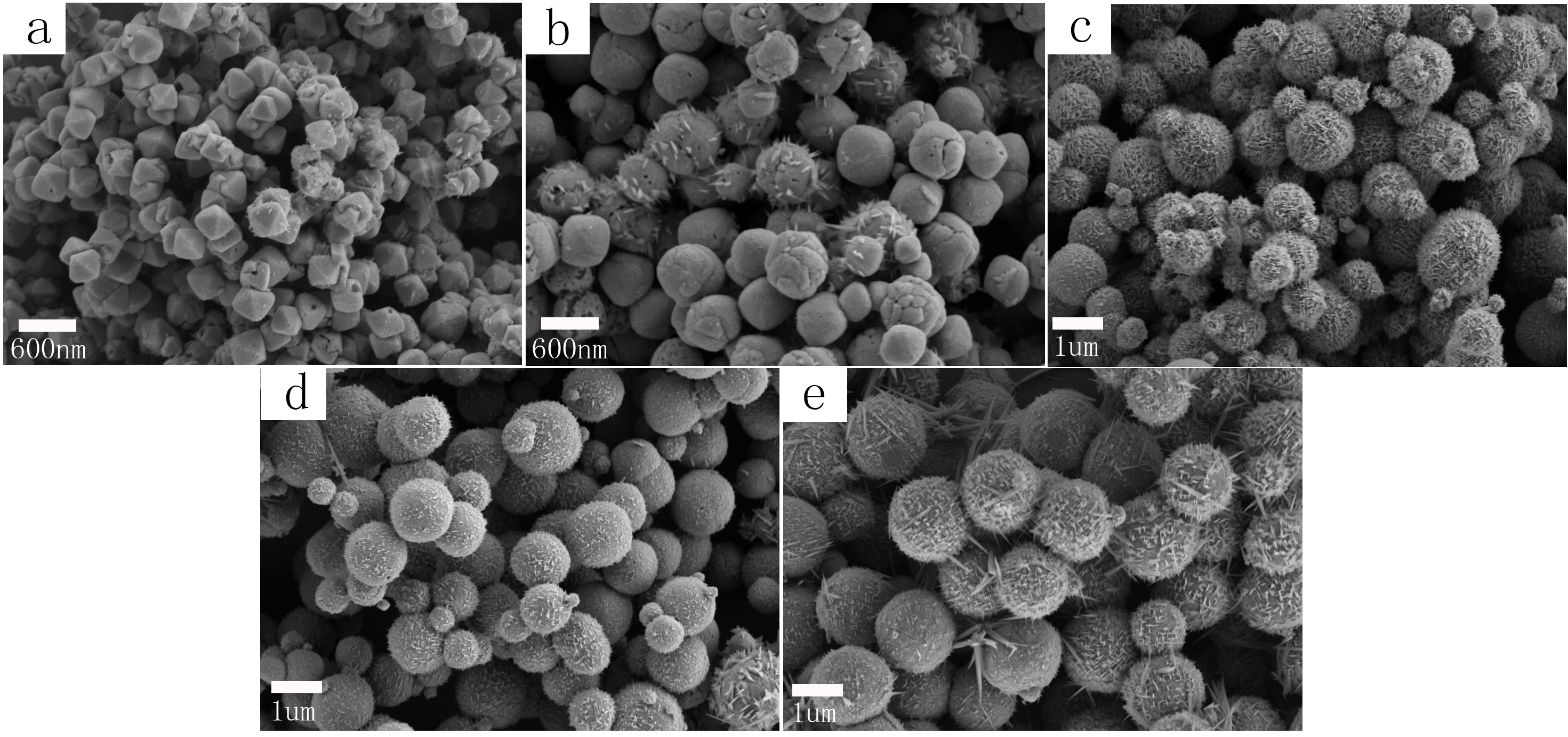


**Supporting Information 5**

SEM images of Cu_2_O samples used in Photocatalytic Activity experiment: **(a),** solid octahedral Cu_2_O showed in fig. 3a; **(b),** hollow sphere Cu_2_O showed in fig. 1a; **(c),** hollow octahedral Cu_2_O (SI1-D3); **(d),** hollow octahedral Cu_2_O showed in fig. 3b; **(e),** hollow octahedral Cu_2_O showed in fig. 1b; **(f),** hollow sphere Cu_2_O showed in fig. 4d.


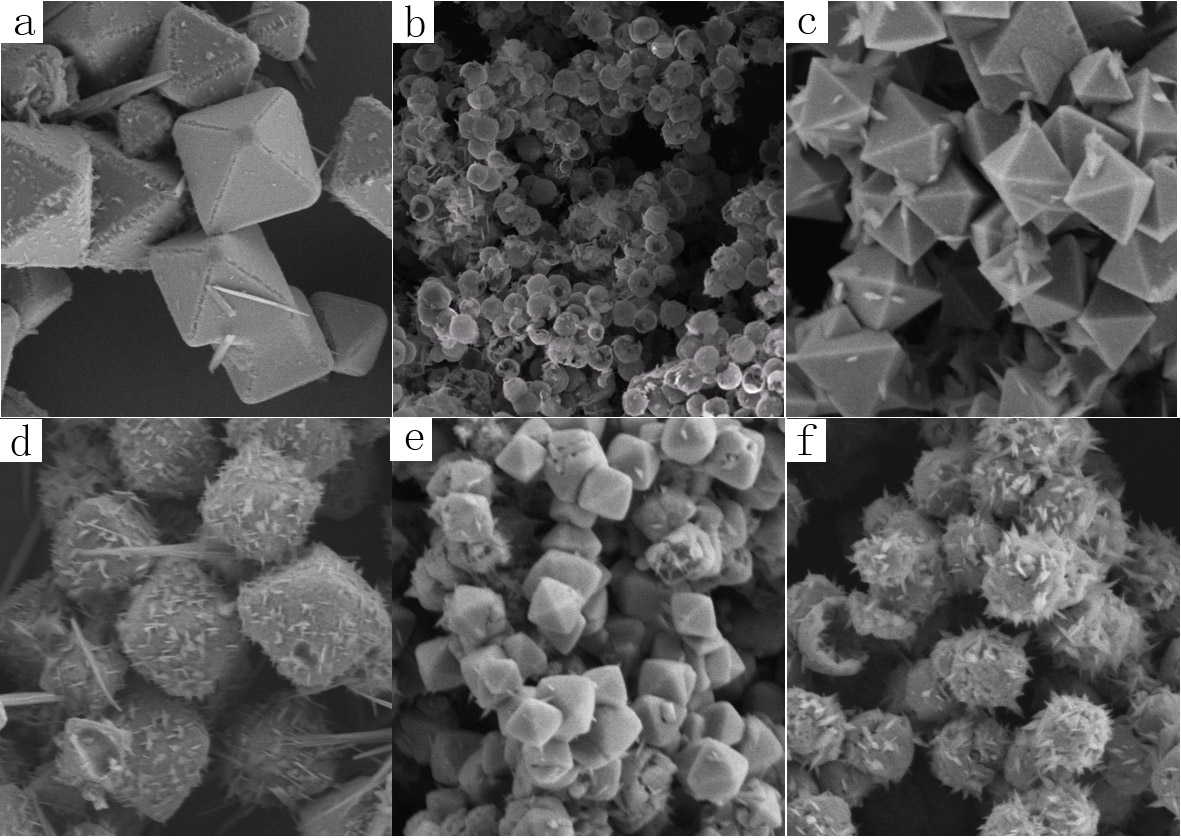


**Supporting Information 6**

Plots of absorbance versus irradiation time of methyl orange and hydrogen peroxide aqueous solution (blank line); Plots of absorbance versus irradiation time of methyl orange aqueous solution in the presence of sphere shaped Cu_2_O sample.


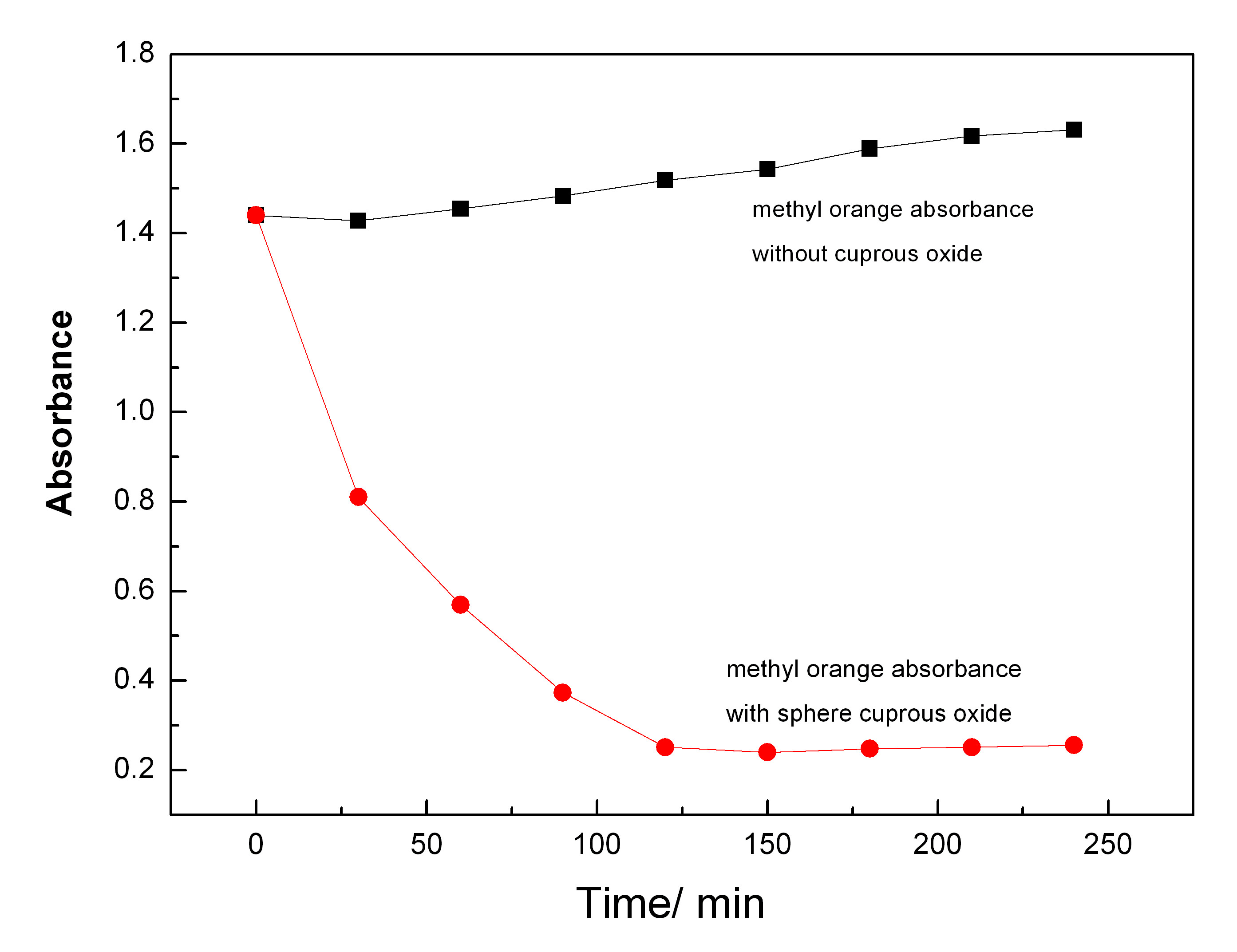

Supplement: Additional file 1 — Supporting information. A document showing supporting information 1 to 6 for facile synthesis of hollow Cu2O octahedral and spherical nanocrystals and their morphology-dependent photocatalytic properties. [file 1556-276X-7-276-S1.doc]
